# Enhancing Psychiatry Residents’ Knowledge and Comfort With Clozapine Prescribing Through Educational Interventions

**DOI:** 10.7759/cureus.108508

**Published:** 2026-05-08

**Authors:** Christopher Stewart, Alexander Hunter, Faith Franzwa, Quan Huynh, Jennifer Stettler, Nauman Ashraf, Dawny M Barnhart

**Affiliations:** 1 Medicine, Kansas City University, Joplin, USA; 2 Psychiatry, Freeman Health System, Joplin, USA

**Keywords:** clozapine education, medical education, medical resident education, psychiatry, quality improvement research

## Abstract

Introduction: Clozapine is the most effective antipsychotic for treatment-resistant schizophrenia but remains underutilized due to safety concerns and monitoring complexity. Recent discontinuation of the Clozapine Risk Evaluation and Mitigation Strategy (REMS) program reduced regulatory barriers but shifted responsibility for monitoring to clinicians. Psychiatry residents may lack adequate training and confidence to prescribe clozapine safely in this new context. The goal of this study is to evaluate the impact of a structured educational intervention on psychiatry residents’ knowledge of clozapine prescribing and management.

Methods: This study was a single-institution quality improvement study using a pre-post design. Participants were psychiatry residents from PGY-1 to PGY-4. Participants underwent a one-hour structured educational session covering clozapine pharmacology, indications, monitoring, adverse effects, and post-REMS management. Outcomes assessed include knowledge assessment scores both before and after the educational session. A paired t-test comparing pre- and post-intervention scores was used to analyze the data.

Results: Ten residents completed both assessments. Mean knowledge scores increased from 69.4% to 82.8% (mean increase 13.4 percentage points (19.3% relative increase); p < 0.05).

Conclusions: A brief, targeted educational intervention was associated with improved psychiatry residents’ knowledge regarding clozapine prescribing and management. Focused resident education may help mitigate barriers to clozapine use, particularly following REMS discontinuation. Incorporating structured clozapine training into residency curricula may promote safer and more appropriate use of clozapine in clinical practice.

## Introduction

Clozapine is a second-generation (atypical) antipsychotic and remains the most efficacious Food and Drug Administration (FDA)-approved treatment for treatment-resistant schizophrenia (TRS) [1]. Although its exact mechanism of action is not fully established, its therapeutic effects are thought to be mediated primarily through antagonism of dopamine D2 and serotonin 5-hydroxytryptamine 2A (5-HT2A) receptors, with additional activity at adrenergic, cholinergic, histaminergic, and other dopaminergic and serotonergic receptors [2]. Clozapine is FDA-approved for TRS and for the reduction of recurrent suicidal behavior in patients with schizophrenia or schizoaffective disorder; it is also used off-label in selected refractory psychiatric and neurologic conditions [3]. TRS is generally defined as persistent clinically significant symptoms despite at least two adequate antipsychotic trials, and consensus criteria further emphasize adequate dose, duration, adherence, and associated functional impairment [4]. Clozapine is considered the gold-standard treatment for TRS because of its superior efficacy compared with other antipsychotics; in the landmark Kane et al. trial, response occurred in 30% of clozapine-treated patients versus 4% of chlorpromazine-treated patients, and meta-analytic data suggest an overall response rate of approximately 40.1% in TRS [1].

Despite this, clozapine remains underutilized because of its serious adverse effect profile, including severe neutropenia, orthostatic hypotension, seizures, myocarditis/cardiomyopathy, gastrointestinal hypomotility, and increased mortality in elderly patients with dementia-related psychosis, as well as the need for baseline and ongoing absolute neutrophil count (ANC) monitoring [5]. Standard initiation requires a baseline ANC of at least 1,500/µL in the general population or at least 1,000/µL in patients with documented benign ethnic neutropenia, with monitoring weekly for the first six months, every two weeks for months 6-12, and monthly thereafter [6]. Dosing is typically initiated at 12.5 mg once or twice daily and titrated gradually to 300-450 mg/day by two weeks as tolerated [6]. Historically, clozapine prescribing in the United States also required participation in the Clozapine Risk Evaluation and Mitigation Strategy (REMS) program, which added administrative requirements including prescriber and pharmacy certification, patient enrollment, ANC reporting, and dispense authorization procedures [7]. In February 2025, the FDA announced that the REMS would be eliminated, and in August 2025, the program was formally removed after determining that it was no longer necessary to ensure safe use and that it could impede patient access to treatment [8]. In the post-REMS era, responsibility for safe prescribing, monitoring, and trainee education rests even more directly with clinicians and training programs [9].

Despite clozapine’s proven efficacy and the removal of REMS-related restrictions, gaps persist in psychiatry residents’ knowledge, prescribing familiarity, and confidence in managing patients treated with clozapine [10]. This likely reflects clozapine’s uniquely complex adverse effect profile and the need for more intensive clinical monitoring than is required for most other antipsychotics. Serious adverse effects include severe neutropenia/agranulocytosis, myocarditis, pericarditis, cardiomyopathy, orthostatic hypotension, bradycardia, syncope, seizures, and severe gastrointestinal hypomotility/constipation; clozapine is also associated with substantial metabolic toxicity, including weight gain, dyslipidemia, and impaired glucose regulation [5,11]. Before initiation, recommended baseline assessment includes complete blood count with ANC, body weight or body mass index, waist circumference, blood pressure, fasting plasma glucose or hemoglobin A1c, lipid profile, bowel-function history, and cardiovascular evaluation, including electrocardiography when clinically indicated [5,11]. Hematologic monitoring is essential because of the risk of agranulocytosis: in the general population, baseline ANC should be at least 1,500/µL prior to initiation, followed by weekly ANC monitoring for the first six months, every two weeks for months 6-12, and monthly thereafter if counts remain within the acceptable range [11]. Ongoing follow-up should also include surveillance for weight change, metabolic abnormalities, constipation and other gastrointestinal symptoms, orthostatic vital signs when indicated, and early signs of myocarditis, particularly during the first weeks of treatment [5,12].

Given the multifaceted monitoring requirements, substantial adverse effect profile, and discontinuation of REMS-mandated safeguards, targeted psychiatry resident education is critical to the safe and effective use of clozapine. This quality improvement (QI) study aimed to evaluate the impact of a structured educational intervention on psychiatry residents’ knowledge of clozapine prescribing and management. By providing focused education on clozapine pharmacology, monitoring, and long-term management, this project seeks to better prepare trainees to appropriately utilize clozapine in clinical practice, thereby improving care and access for patients with TRS.

This study was previously presented as a poster at the Kansas City University Research Symposium in Joplin, Missouri, in April 2025.

## Materials and methods

Study design

This project was conducted as a single-institution QI study using a single-arm pre-post intervention design. Psychiatry residents completed a baseline knowledge assessment immediately prior to participation in a structured educational session on clozapine prescribing. Following completion of the one-hour educational intervention, participants completed the same assessment to evaluate changes in knowledge.

The primary outcome was the change in knowledge assessment scores following the intervention. No identifiable participant data were collected. Participation was voluntary, and the project met criteria for institutional review board exemption per the Freeman Health System policy.

Setting and participants

The survey was conducted at a single psychiatry residency program at Freeman Health System in March 2025. Participants included psychiatry residents across all postgraduate training levels (PGY-1 to PGY-4). Participation was voluntary, and informed consent was collected from all participants. Participants were recruited using a convenience sampling approach, consisting of psychiatry residents who were available and voluntarily attended the educational session. A total of 11 residents participated in the study. One resident did not complete the survey as they were performing clinical responsibilities and were unable to attend the educational session.

Inclusion criteria consisted of all psychiatry residents enrolled in the Freeman Health System Psychiatry Residency Program (PGY-1 through PGY-4) who attended the educational session and consented to participate. Exclusion criteria included residents who were unable to attend the educational session or did not complete both the pre- and post-intervention assessments.

Educational intervention

A structured, resident-led, one-hour educational session focused on clozapine prescribing and management was conducted. The session was performed at one time, and all participants were physically present. The content of the educational session included the mechanism of action and pharmacology, FDA-approved and off-label indications, dosing and titration guidelines, side effect profile and contraindications, monitoring requirements and recommendations before and after discontinuation of the Clozapine REMS program, management of adverse effects, and long-term monitoring strategies. The content was delivered in a lecture format.

Data collection

A 16-question assessment was administered immediately before and after the intervention (Appendix). The assessment consisted of 11 multiple-choice questions and five open-ended questions. Knowledge assessment scores were calculated by assigning one point for each correct response. Multiple-choice questions were scored as correct or incorrect, and open-ended responses were evaluated based on predefined correct answers. Total scores were summed and converted to percentages based on the total number of questions. The clozapine QI assessment was developed by the study authors for the purposes of this project and was not adapted from a previously validated instrument. The questions assessed the indications for clozapine, dosing and monitoring, adverse effects and management, and practical considerations following REMS discontinuation. Responses were de-identified and used for analysis. Demographic data are limited to postgraduate education levels to preserve confidentiality.

Statistical analysis

Descriptive statistics were used to summarize participant characteristics and assessment performance. Continuous variables are reported as means and standard deviations (SDs). Pre- and post-intervention knowledge scores were compared using paired t-tests to evaluate changes following the educational intervention. The paired t-test was conducted using pre-intervention minus post-intervention scores, resulting in a negative t-value reflecting improvement following the intervention. Percent change in scores was calculated to quantify improvement. A two-tailed significance level of α = 0.05 was used. Statistical analyses were performed using a TI-84 calculator.

## Results

Demographics

A total of 11 psychiatry residents across postgraduate years PGY-1 through PGY-4 participated in the educational intervention. Ten residents completed both the pre- and post-intervention assessments. One resident was unable to complete the post-intervention survey due to concurrent clinical responsibilities. Demographics of the participants are found in Table 1.

**Table 1:** Resident demographics. PGY: postgraduate year

Year of residency	Number of participants	Percent
PGY-1	4	40%
PGY-2	3	30%
PGY-3	0	0%
PGY-4	3	30%
Total	10	100%

Knowledge assessment outcomes

**Figure 1:**
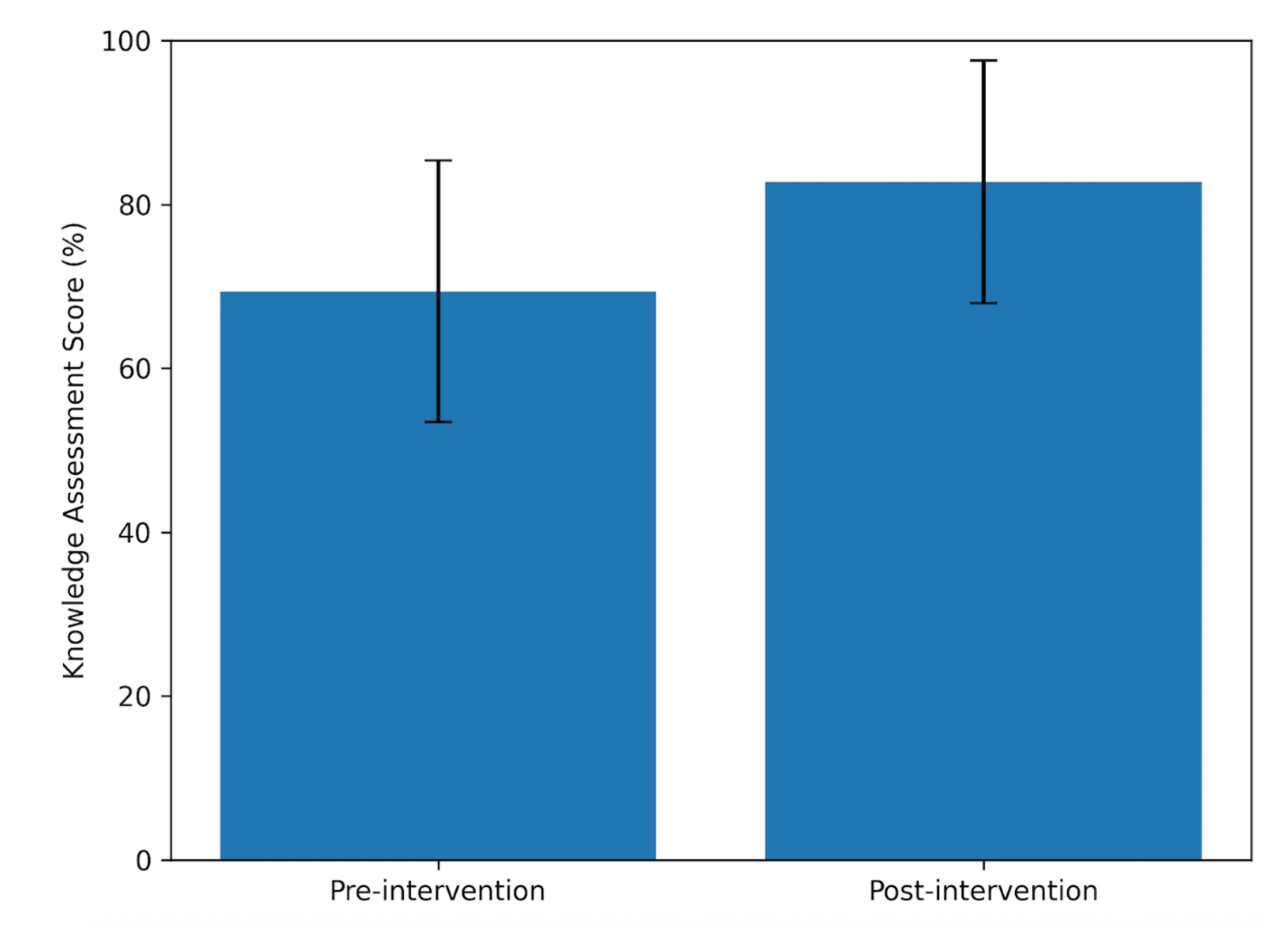
Pre- and post-intervention knowledge assessment scores. The bar graph shows mean knowledge assessment scores (%) before and after the educational intervention. Mean scores increased from approximately 69% pre-intervention to 83% post-intervention, indicating an overall improvement in knowledge following the intervention. Error bars represent standard deviations, illustrating the variability of scores within each assessment period.

Pre-intervention knowledge assessment scores averaged 69.4% (SD = 15.98), whereas post-intervention scores averaged 82.8% (SD = 14.82), representing a mean increase of 13.4 percentage points (19.3% relative increase). A paired t-test demonstrated a statistically significant improvement in test scores following the educational intervention (t(9) = −2.86, p < 0.05). These findings can be seen in Figure 1.

Table 2 summarizes the statistical comparison of pre- and post-intervention knowledge scores.

**Table 2:** Statistical comparison of pre- and post-intervention knowledge scores.

Outcome	Mean difference	% change	t-value	p-value
Knowledge score (%)	13.4	19.30%	-2.86	<0.05

## Discussion

Summary of key findings

This study demonstrated that a short educational course can significantly increase knowledge about clozapine and reduce barriers to its use. Following the lecture, residents showed improved knowledge scores related to prescribing clozapine, a critical skill following the discontinuation of REMS requirements, which has placed greater responsibility on individual physicians. Given clozapine’s unique efficacy paired with its potential for serious complications, ensuring prescribers are well-informed is essential. This model of targeted, short-term education could also be applied to other complex medications throughout residency to enhance patient care.

These findings are consistent with prior studies evaluating educational interventions in psychiatry and other medical specialties, which have demonstrated that brief, targeted teaching sessions can significantly improve clinician knowledge and preparedness for managing complex medications [13]. Similar studies have shown improvements in knowledge and prescribing practices following structured educational modules focused on high-risk or underutilized therapies [14]. Our results align with this body of literature, suggesting that even short, focused interventions can effectively address knowledge gaps related to clozapine prescribing.

Importance of clozapine education

This is important because underprescription leads to increased hospitalizations, higher healthcare costs, and worse patient outcomes [12]. Clozapine use is also associated with potentially serious adverse effects, including agranulocytosis (neutropenia), myocarditis, seizures, and metabolic complications, which necessitate careful monitoring and contribute to clinician hesitancy in prescribing [11]. Barriers that prevent clinician prescription of clozapine include a lack of experience, concerns about side effects, and insufficient training [15]. This study suggests that structured education may help address these barriers by educating psychiatry residents to prescribe clozapine appropriately and safely.

Clozapine is consistently identified as the most effective antipsychotic for TRS, yet it remains substantially underutilized [16]. Multiple studies demonstrate that only a minority of eligible patients ultimately receive clozapine, with prescription rates far below the estimated prevalence of treatment resistance [12,16,17]. In the United States, approximately 20%-30% of patients with schizophrenia meet criteria for treatment-resistant illness and are therefore candidates for clozapine; however, fewer than 5% of patients with schizophrenia are treated with clozapine, highlighting a significant gap between guideline-supported therapy and real-world practice [12,16,17]. This underutilization is clinically meaningful, as delayed or absent clozapine initiation is associated with increased hospitalizations, higher healthcare costs, and poorer long-term outcomes [18,19]. Documented barriers to clozapine prescribing include limited clinician experience, concerns regarding adverse effects, administrative burden, and insufficient formal training [4,9]. This study sought to address these modifiable barriers by providing structured education to psychiatry residents aimed at improving knowledge and safe prescribing practices.

This short educational course was associated with improved knowledge as measured by pre- and post-surveys. Residents started with moderate baseline knowledge identified by the pre-survey, which helped highlight important knowledge gaps. The educational session improved understanding of clozapine pharmacology and necessary monitoring. Early exposure to this education helps develop proficiency in managing complex medications under the supervision of attending physicians. Gaining experience during residency helps residents become comfortable monitoring adverse effects and prescribing safely, which then increases the likelihood they will use these medications appropriately as attendings.

Clinical implications

Appropriate and timely use of clozapine is associated with meaningful clinical benefits including higher remission rates, reduced suicidality, fewer hospitalizations, and improved quality of life [18,19]. Improving clinician knowledge, as addressed by this educational intervention, may reduce delays in clozapine initiation. This could, in turn, help to decrease hospitalization rates and lengths of stay, as well as reduce unnecessary medication trials and polypharmacy [12].

Necessary monitoring for clozapine includes routine laboratory evaluation for neutropenia, myocarditis, and liver damage [6]. It is outside the scope of this paper to go through all potential side effects and adverse effects; however, an improved understanding of monitoring protocols reduces the risk of missed side effects. Residents trained early may be more consistent in ordering baseline labs, titration labs, and long-term metabolic management. Additionally, improved knowledge of side effect recognition and management may reduce unnecessary discontinuations of clozapine.

An additional clinical relevance of clozapine education is regarding the recent discontinuation of the Clozapine REMS program [8]. Under REMS, patients were required to be enrolled in a federal registry, pharmacies needed special certification to dispense clozapine, and clinicians were required to submit regular ANC monitoring data to a centralized database. This system standardized safety monitoring but also created a substantial administrative burden for both clinicians and patients [7]. With the removal of REMS, some of these logistical barriers have been eliminated, making it easier to initiate clozapine for patients who need it. At the same time, the responsibility for ensuring appropriate laboratory monitoring and ongoing safety assessment now rests entirely on individual clinicians. This shift underscores the importance of equipping residents with the knowledge required to safely prescribe and monitor clozapine without reliance on a centralized system.

Strengths, limitations, and future directions

A notable strength of this project was the inclusion of residents across all training levels (PGY-1 to PGY-4), allowing for representation of learners with varying levels of clinical experience. However, several limitations must be acknowledged. The questionnaire used in this study was self-developed and not formally validated, which may limit the reliability and generalizability of the findings. The sample size (n = 10) is also small and limited to a single institution, which may impact the generalizability of the findings to larger academic centers. Additionally, this study measured immediate post-intervention gains; it remains unclear whether this knowledge is retained long-term or if it translates into a tangible increase in clozapine prescriptions in our outpatient clinics. Finally, there were no PGY-3 participants included in the study.

Future research should move beyond objective knowledge testing to evaluate behavioral outcomes. Prospective studies could assess whether residents who undergo this training are more likely to initiate clozapine or demonstrate higher adherence to monitoring guidelines compared to those who do not. As psychiatry transitions into the post-REMS era, developing a standardized, case-based education module-incorporating management of high-risk adverse effects such as agranulocytosis and myocarditis-will be essential for maintaining standard-of-care treatment in psychiatry.

## Conclusions

Clozapine remains underused despite superior efficacy, with prescribing challenges heightened by REMS discontinuation and the complexity of monitoring requirements. This QI study suggests that a brief educational intervention may improve psychiatry residents’ knowledge regarding clozapine prescribing and management. Improved resident preparedness may reduce delays in clozapine initiation, inappropriate discontinuation, and safety risks. As external safeguards like REMS are removed, clinician and resident education becomes a critical patient safety mechanism. Residency programs should consider integrating standardized clozapine education early and longitudinally. Similar educational models could be applied to other high-risk, high-benefit psychiatric medications. Targeted clozapine education may represent a practical strategy to improve resident competency and expand access to an underutilized and potentially life-changing treatment for patients with TRS.

## Appendices

**Table 3:** Pre- and post-assessment questions.

Question No.	Question	Response type
1	Clozapine primarily acts as an antagonist at which receptor, contributing to its antipsychotic effects? ☐ D2 receptor ☐ D4 receptor ☐ 5-HT2A receptor ☐ NMDA receptor	Multiple choice
2	Clozapine is FDA-approved for which of the following conditions? ☐ Treatment-resistant schizophrenia ☐ Parkinson’s disease psychosis ☐ Schizoaffective disorder ☐ Reduction of suicidal behavior in schizophrenia	Multiple choice
3	Which of the following is true regarding clozapine-induced agranulocytosis? ☐ It typically occurs within the first week of treatment ☐ It requires immediate discontinuation of clozapine if ANC drops below 1,500/μL ☐ The risk is highest in the first 6 months of therapy ☐ The incidence rate is greater than 10%	Multiple choice
4	How frequently is ANC monitoring required during the first 6 months of clozapine treatment? ☐ Weekly ☐ Biweekly ☐ Monthly ☐ Only at initiation and then every 6 months	Multiple choice
5	Which of the following side effects is clozapine least likely to cause? ☐ Sialorrhea ☐ Metabolic syndrome ☐ Tardive dyskinesia ☐ Myocarditis	Multiple choice
6	What is the most appropriate initial test to evaluate for clozapine-induced myocarditis in a symptomatic patient? ☐ Echocardiogram ☐ Serum troponin and C-reactive protein (CRP) levels ☐ Electrocardiogram (ECG) ☐ Cardiac MRI	Multiple choice
7	Which of the following drugs is most likely to increase serum levels of clozapine? ☐ Phenytoin ☐ Rifampin ☐ Fluoxetine ☐ Carbamazepine	Multiple choice
8	How does smoking affect clozapine metabolism, and what is the clinical implication? ☐ Smoking inhibits clozapine metabolism, leading to toxicity ☐ Smoking induces clozapine metabolism, potentially reducing efficacy ☐ Smoking has no significant effect on clozapine levels ☐ Smoking increases the risk of agranulocytosis	Multiple choice
9	Why must clozapine dosage be titrated slowly upon initiation? ☐ To prevent seizures ☐ To minimize the risk of orthostatic hypotension ☐ To reduce the likelihood of gastrointestinal side effects ☐ To avoid QT prolongation	Multiple choice
10	When is it appropriate to consider rechallenging a patient with clozapine after a previous discontinuation due to side effects? ☐ After any side effect resolves ☐ If agranulocytosis occurred but the patient is now stable ☐ For mild myocarditis after 6 months ☐ After neutropenia resolves and the risk-benefit analysis supports rechallenge	Multiple choice
11	Case-based question: A 30-year-old female with treatment-resistant schizophrenia on clozapine presents with flu-like symptoms, fever, elevated CRP, and elevated troponin. What serious adverse effect must be considered, what is the significance of these findings, and what immediate steps should be taken regarding clozapine therapy?	Open-ended
12	Case-based question: A 62-year-old patient with schizophrenia and dementia-related psychosis on clozapine develops confusion, orthostatic hypotension, and falls while taking a benzodiazepine. What risks are exacerbated, how do benzodiazepines contribute, and how should fall risk be mitigated?	Open-ended
13	Case-based question: A 48-year-old male with a baseline ANC of 1,400 cells/μL is being considered for clozapine. How would initiation be approached, what monitoring is required, and when would clozapine be held or discontinued?	Open-ended
14	Case-based question: A 35-year-old male on clozapine is admitted for a seizure with eosinophilia. What risk factors may have contributed, what does eosinophilia indicate, and would clozapine be resumed?	Open-ended
15	Case-based question: A 52-year-old female on clozapine presents with tachycardia, tremors, blurry vision, and constipation. What pharmacologic mechanisms explain these symptoms, and how would management be adjusted?	Open-ended
16	What year of training are you? ☐ PGY-1 ☐ PGY-2 ☐ PGY-3 ☐ PGY-4	Multiple choice
